# Optimization of sepsis therapy based on patient-specific digital precision diagnostics using next generation sequencing (DigiSep-Trial)—study protocol for a randomized, controlled, interventional, open-label, multicenter trial

**DOI:** 10.1186/s13063-021-05667-x

**Published:** 2021-10-18

**Authors:** Thorsten Brenner, Annabell Skarabis, Philip Stevens, Jennifer Axnick, Peter Haug, Silke Grumaz, Thomas Bruckner, Steffen Luntz, Oliver Witzke, Mathias W. Pletz, Thomas M. Ruprecht, Ursula Marschall, Sibel Altin, Wolfgang Greiner, Marc Moritz Berger, for the TIFOnet Critical Care Trials Group

**Affiliations:** 1 Department of Anesthesiology and Intensive Care Medicine, Essen University Hospital, University Duisburg-Essen, Hufelandstr. 55, 45147 Essen, Germany; 2 Noscendo GmbH, Königstraße 34, 47198 Duisburg, Germany; 3 Institute of Medical Biometry, University of Heidelberg, Im Neuenheimer Feld 130.3, 69120 Heidelberg, Germany; 4 Coordination Centre for Clinical Trials (KKS), Ruprecht-Karls-University, Im Neuenheimer Feld 130.3, 69120 Heidelberg, Germany; 5 Department of Infectious Diseases, University Hospital Essen, University Duisburg-Essen, Hufelandstr. 55, 45147 Essen, Germany; 6 Center for Infectious Diseases and Infection Control, Jena University Hospital, Erlanger Allee 101, 07740 Jena, Germany; 7 Techniker Krankenkasse, Bramfelder Str. 140, 22305 Hamburg, Germany; 8 BARMER, Lichtscheider Str. 89, 42285 Wuppertal, Germany; 9 AOK Rheinland/Hamburg, Kasernenstr. 61, 40213 Düsseldorf, Germany; 10 Health Economics and Health Care Management, Bielefeld University, P.O. Box 10 01 31, 33501 Bielefeld, Germany

**Keywords:** Sepsis, Bacteremia, Blood culture, Next generation sequencing, Digital precision diagnostics, Desirability of Outcome Ranking/Response Adjusted for Duration of Antibiotic Risk (DOOR/RADAR) score

## Abstract

**Background:**

Sepsis is triggered by an infection and represents one of the greatest challenges of modern intensive care medicine. With regard to a targeted antimicrobial treatment strategy, the earliest possible pathogen detection is of crucial importance. Until now, culture-based detection methods represent the diagnostic gold standard, although they are characterized by numerous limitations. Culture-independent molecular diagnostic procedures represent a promising alternative. In particular, the plasmatic detection of circulating, cell-free DNA by next-generation sequencing (NGS) has shown to be suitable for identifying disease-causing pathogens in patients with bloodstream infections.

**Methods:**

The *DigiSep-Trial* is a randomized, controlled, interventional, open-label, multicenter trial characterizing the effect of the combination of NGS-based digital precision diagnostics with standard-of-care microbiological analyses compared to solely standard-of-care microbiological analyses in the clinical picture of sepsis/septic shock. Additional anti-infective expert consultations are provided for both study groups. In 410 patients (*n* = 205 per arm) with sepsis/septic shock, the study examines whether the so-called DOOR-RADAR (Desirability of Outcome Ranking/Response Adjusted for Duration of Antibiotic Risk) score (representing a combined endpoint including the criteria (1) intensive/intermediate care unit length of stay, (2) consumption of antibiotics, (3) mortality, and (4) acute kidney injury (AKI)) can be improved by an additional NGS-based diagnostic concept. We also aim to investigate the cost-effectiveness of this new diagnostic procedure. It is postulated that intensive/intermediate care unit length of stay, mortality rate, incidence of AKI, the duration of antimicrobial therapy as well as the costs caused by complications and outpatient aftercare can be reduced. Moreover, a significant improvement in patient’s quality of life is expected.

**Discussion:**

The authors´ previous work suggests that NGS-based diagnostics have a higher specificity and sensitivity compared to standard-of-care microbiological analyses for detecting bloodstream infections. In combination with the here presented *DigiSep*-Trial, this work provides the optimal basis to establish a new NGS-driven concept as part of the national standard based on the best possible evidence.

**Trial registrations:**

DRKS-ID DRKS00022782. Registered on August 25, 2020

ClinicalTrials.govNCT04571801. Registered October 1, 2020

**Supplementary Information:**

The online version contains supplementary material available at 10.1186/s13063-021-05667-x.

## Background

With an annual global incidence of nearly 50 million cases and up to 11 million deaths per year (accounting for 20% of total mortality worldwide), sepsis remains an ongoing challenge in intensive care medicine [1]. Sepsis is defined as life-threatening organ dysfunction resulting from a dysregulated host response to infection. Due to relevant diagnostic limitations, microbial detection has not become an obligatory part of recent sepsis definitions [2]. Besides viruses, fungi, and parasites, sepsis-inducing infections are primarily caused by bacteria. In parallel and complementary to focus control measures, current sepsis guidelines therefore recommend an earliest possible empiric antibiotic therapy (preferably within 1 h) following sepsis diagnosis [3, 4]. Up to now, culture-based diagnostic procedures (e.g., blood cultures) are defined as diagnostic gold standard, although they are associated with relevant limitations (such as high time requirements, low sensitivity due to concomitant antibiotic therapy or being susceptible to microbial contaminations) [3–7]. Accordingly, septic patients are frequently confronted with an antimicrobial overtreatment, resulting in an increased selection of multi-drug resistant pathogens. Moreover, an inadequate or prolonged use of antibiotics is associated with a high risk for antibiotics-related toxicity, leading to short as well as long-term complications (such as acute kidney injury (AKI) with a high risk for persisting dependency on renal replacement therapy (RRT)) in septic patients. Apart from its impact on the initial anti-infective treatment strategy, the use of a more targeted pathogen diagnostic may help to avoid sepsis-associated organ dysfunctions in the early disease course and has a cross-sectoral influence on aftercare measures of affected patients [8]. The authors’ previous work suggests that next generation sequencing (NGS)-based diagnostics for pathogen identification might be a promising alternative due to its higher specificity and sensitivity compared to culture-based methods in bloodstream infections [9–11]. However, a head-to-head clinical study comparing the efficacy of this new NGS-based approach (as part of a comprehensive diagnostic workup in patients with suspected or proven sepsis/septic shock) with standard-of-care microbiological analyses is lacking. The aim of the here presented randomized, controlled, interventional, open-label, multicenter trial is therefore to characterize the effect of the combination of NGS-based digital precision diagnostics with culture-based standard diagnostics compared to solely standard-of-care microbiological analyses in the clinical picture of sepsis/septic shock.

## Methods

### Aim, design, and study setting

The primary objective of *DigiSep* is to investigate whether the so-called DOOR-RADAR (Desirability of Outcome Ranking/Response Adjusted for Duration of Antibiotic Risk) score can be significantly improved by application of an additional NGS-based diagnostic concept. The study is a randomized, controlled, interventional, open-label, multicenter trial on medical as well as surgical intensive care units (ICUs) of maximum care hospitals throughout the *Translational Intensive Care Research Network on Organ Dysfunction* (TIFOnet) in Germany. Coordinating center of the study is the Department of Anesthesiology and Intensive Care Medicine, University Hospital Essen. Noscendo GmbH, Duisburg, is responsible for NGS-based measurements in plasma samples of septic patients. Optional expert consultations are provided by experienced infectiologists of the Department of Infectious Diseases, University Hospital Essen. Project management is performed by the Coordination Centre for Clinical Trials (KKS), University of Heidelberg. Data management and statistical analysis are provided by the Institute for Medical Biometry (IMBI), University of Heidelberg. Statutory health insurance data will be provided by three different German statutory health insurers (AOK Rheinland-Hamburg, BARMER, Techniker Krankenkasse). Health economic analyses will be performed by the chair of Health Economics and Health Care Management, Bielefeld University. The study protocol follows the Standard Protocol Items: Recommendations for Interventional Trials (SPIRIT) guidelines (Supplemental File 1).

### Characteristics of participants

Patients with sepsis or septic shock according to current sepsis definitions (Sepsis-3) [2] with a sepsis onset < 24 h are eligible for study inclusion, independent of their insurance allegiance. A summary of all inclusion and exclusion criteria for participants is given in Table 1. The data of all these patients will be used for the evaluation of the study’s primary endpoint (i.e., the DOOR-RADAR score). For the analyses of several secondary endpoints, however, only patients of the three German health insurers involved in the study can be included. These are expected to be about 30% of all patients. Of note, also with the expected reduced number of patients (i.e., with about 135 patients), these secondary outcomes are sufficiently powered.

**Table 1:** Inclusion and exclusion criteria of *DigiSep*-trial

**Inclusion criteria**	
Age ≥ 18 years	
Informed consent	
Sepsis *or* septic shock (with an onset ≤ 24 h)	
**Exclusion criteria**	
Age < 18 years	
Refusal to give consent	
Patient will probably be discharged from the ICU/IMC within the first 72 h following inclusion	
Palliative treatment intent	
Clinician is not committed to aggressive treatment	
Death is deemed imminent and inevitable	
Patients who had previously been included but are readmitted to the ICU/IMC during the same hospitalization will not be included a second time.	

Abbreviations: *ICU* intensive care unit; *IMC* intermediate care unit

### Randomization process and interventions

Eligible patients will be randomized (1:1) into one of the two different study arms (CG, control group/IG, intervention group) using block randomization with randomized block length. The randomization is done stratified by study center using an internet-based tool (URL: www.randomizer.at). The randomization is blinded for the patient, blinding of the attended physician is not possible. All included patients will be treated according to current sepsis guidelines [3, 4]. Pathogen diagnostics in patients of the CG will be done with standard-of-care microbiological analyses followed by optional expert consultations, whereas in patients of the IG, a comprehensive concept of digital precision diagnostics (including NGS-based diagnostics, standard-of-care microbiological analyses, and optional expert consultations) will be provided.

### Description of the used interventions

#### Standard-of-care microbiological analyses

Standard-of-care microbiological analyses of potential pathogens in the different specimens (also including rare pathogens of opportunistic infections in immuno-compromised patients such as *Aspergillus* spp., *Mucorales*, *P. jirovecii*, *C. neoformans*, etc*.*) will be performed according to the usual practice in each participating institution.

#### Data analysis and result communication using next-generation sequencing (NGS)

NGS-based measurements will be performed by Noscendo GmbH (Noscendo GmbH, Koenigstraße 34, 47198 Duisburg, Germany) in its laboratories at Gerhard-Kindler-Straße 13, 72770 Reutlingen, Germany. Transfer of samples to Noscendo GmbH will be performed at room temperature by a medical logistics integrator coordinated by Noscendo GmbH and called on demand. In detail, plasma samples for NGS will be prepared at Noscendo GmbH from cfDNA stabilizing tubes (Cell-Free DNA BCT CE by Streck, La Vista, NE, USA) by centrifugation. Nucleic acids will be isolated from the freshly prepared plasma with the QIAsymphony DSP Circulating DNA Kit (Qiagen, Hilden, Germany) on the QIAsymphony SP instrument. Adequate controls will accompany all laboratory and sequencing procedures. The cfDNA will be quantified with the Qubit 1x dsDNA HS Assay Kit (Thermo Fischer Scientific, MA, USA), and quality will be assessed by the High Sensitivity DNA Kit (HS NGS Fragment Kit (1–6000 bp)) on a 5200 Fragment Analyzer System (Agilent, Santa Clara, CA, USA). DNA library preparation will be performed from 1 ng cfDNA input and DNA libraries will be sequenced using a NextSeq550 (Illumina, San Diego, CA, USA) with 75 bp read length in single end mode. Sequencing depth of the samples will be 25 million single end reads minimum, per sample. Raw sequencing data is subjected to various QC controls comprising, PHRED score filtering, adapter trimming, complexity filtering as well as k-mer-based contamination screening. To pass the quality filter, read quality needs to surpass a Phred score of 20 and achieve a minimal length of 50 bp after quality control. All data generated will be analyzed using Noscendo’s CE-IVD platform DISQVER®. Noscendo’s DISQVER® comprises a curated microbial genome database of over 16.000 microbial species covering more than 1.500 pathogens and can detect bacteria, DNA-viruses, fungi, and parasites in one single assay while differentiating contamination, commensals from infective agents. Results of the DISQVER® test will be made available to the commissioning physician via the digital communications solution of Noscendo (NOS-portal).

#### Facultative expert consultations

In terms of an anti-infective stewardship, facultative expert consultations are available for patients in both study groups. However, they are particularly recommended for patients of the IG group (where results of NGS-based diagnostics are made available to the commissioning physician), if NGS-based digital precision diagnostics and standard-of-care microbiological analyses are contradictory. The characteristics and results of consultations in both groups will be monitored and described in detail in the final publication. Because the involvement of the expert group is possible for all patients, their final effect on patient’s outcome is expected to be comparable between both study groups.

### Participant timeline, data collection, and sample handling

#### Data collection

As part of the study, patients´ baseline data is collected once at the time of sepsis diagnosis (= baseline): patient demographics (e.g., age, sex), date and time of hospital and ICU admission, admission source (e.g., emergency department, outpatient clinic/referral, operating room, post-anesthesia care unit, and other hospital units), major comorbid conditions, immune status (host factors predisposing for an immunodeficiency according to [12]; Supplemental File 2), site of suspected or confirmed infection, antimicrobial course prior to study enrolment, surgery/procedures for suspected site of infection prior to enrolment, and Sequential Organ Failure Assessment (SOFA) score (Supplemental File 3). Clinical data collection is carried out at the time of sepsis diagnosis (= baseline), 3, 7, 14, and 28 days later. All laboratory parameters will be measured according to the local standard. The outcome evaluation takes place 28 days after the onset of sepsis. Clinical data collection during admission will include pertinent laboratory data, use of mechanical ventilation, and antimicrobial/antibiotic therapy including duration of therapy, and date therapy will be initiated and discontinued. Vasoactive drug therapy, renal replacement therapy, surgical and other procedures for diagnosis/treatment of infection, radiological testing for diagnosis/evaluation of potential infection, indwelling vascular access devices, and vital status will also be recorded. Discharge data will include the date of discharge (ICU/IMC, and hospital), discharge destination (general hospital ward, skilled nursing facility, and home), and vital status at discharge (survival/death). Finally, quality of life (QoL) of all participating patients will be assessed at onset, 90 and 180 days after sepsis onset using the VR-36 questionnaire [13].

#### Sample collection and sample handling

Culture-based diagnostics include the guideline compliant collection of 2 blood culture sets (2 x aerobic/2 x anaerobic) at baseline and 3 days later. At the same time, blood samples are obtained for NGS-based pathogen diagnostics. Additional sampling for NGS-based diagnostics can be made up to day 14 after the baseline visit or whenever the attending physician establishes a clinical indication for collecting further blood cultures. Routine microbiological findings from other biological samples (e.g., surgical swabs, drainage secretions, tracheal secretions, tissue samples) are included in the evaluation if these were collected within 3 days before or after the extraction of blood samples for NGS-based diagnostics. The study-related burden on the individual study patient includes a total of 10–20 ml whole blood for NGS-based diagnostics. The minimum total volume, therefore, amounts to the collection of 10 ml whole blood within the first 14 days after the onset of sepsis. The sampling takes place with the collection of the blood cultures or within the framework of the daily routine blood samples, so that no further venous punctures are required. Infection parameters such as procalcitonin (PCT) are also carried out within the framework of daily regular blood collection. The same principle applies to the collection of blood cultures which are routinely obtained as part of standard diagnostics in patients with suspected or proven sepsis. The required blood volume of 40 ml whole blood (each two sets of 2 x aerobic/2 x anaerobic = 4 × 10 ml = 40 ml) therefore does not represent an additional study-related burden. A further burden for the patient regarding invasive procedures or examinations is not expected.

### Outcomes

#### Primary outcomes

*DigiSep* primarily evaluates whether the so-called DOOR-RADAR (Desirability of Outcome Ranking/Response Adjusted for Duration of Antibiotic Risk) score can be significantly improved by application of an additional NGS-based diagnostic concept [14] (Table 2).

**Table 2:** Primary and secondary outcomes of *DigiSep*-trial

Outcomes	Data source	Timepoint/frame
Primary outcomes		
DOOR/RADAR score	eCRF	28 days
Secondary outcomes		
Long term mortality	Statutory health insurance data, eCRF	90, 180 days
Degree of organ dysfunction/failure: • Duration of mechanical ventilation • Length of time until shock resolution • Ongoing RRT-dependency	eCRF	28, 90, 180 days
Hospital length of stay	Statutory health insurance data, eCRF	28, 90, 180 days
Cumulative need for anti-infective drugs	Statutory health insurance data, eCRF	28, 90, 180 days
Beginning of a targeted anti-infective treatment regimen	eCRF	28 days
Resource utilization (outpatient and inpatient)	Statutory health insurance data	28, 90, 180 days
Duration and costs of incapacity to work	Statutory health insurance data	28, 90, 180 days
Direct medical costs (outpatient and inpatient)	Statutory health insurance data	28, 90, 180 days
Quality-of-life (QoL)	VR-36 questionnaire	0, 90, 180 days

Abbreviations: *RRT* renal replacement therapy; *QoL* quality of life; *eCRF* electronic case report from; *DOOR/RADAR score* Desirability of Outcome Ranking/Response Adjusted for Duration of Antibiotic Risk score

The DOOR-RADAR score represents a combined clinical endpoint. While the DOOR component reflects the overall clinical outcome (OCO) consisting of intensive/intermediate care unit length of stay (ICU/IMC-LOS), mortality, and RRT-dependent AKI, the RADAR component reflects the duration of anti-infective treatment. Both components are combined to yield the DOOR-RADAR score, which is a numerical value and calculated as follows:

$$ \mathrm{DOOR}-\mathrm{RADAR}\ \mathrm{score}\ \left\{\begin{array}{c}=\mathrm{OCO}+\left[\frac{\mathrm{number}\ \mathrm{of}\ \mathrm{days}\ \mathrm{with}\ \mathrm{anti}-\mathrm{infective}\ \mathrm{treatment}\kern0.5em }{\max .\kern0.5em \mathrm{number}\ \mathrm{of}\ \mathrm{days}\ \mathrm{with}\ \mathrm{anti}-\mathrm{infective}\ \mathrm{treatment}\kern0.5em +1\ \mathrm{day}}\right],\mathrm{if}\ \mathrm{OCO}\le 4\\ {}=5\kern28em ,\mathrm{if}\ \mathrm{OCO}=5\end{array}\right. $$

with the OCO being calculated as

1 point: Survival* + ICU/IMC LOS < 12 days without AKI**

2 points: Survival* + ICU/IMC LOS < 12 days with AKI**

3 points: Survival* + ICU/IMC LOS ≥ 12 days without AKI**

4 points: Survival * + ICU/IMC LOS ≥ 12 days with AKI**

5 points: Death of the patient*

(* at/within 28 days; ** with the need for RRT).

Using the DOOR-RADAR score as primary endpoint, it should be delineated whether a patient of the IG has a higher probability for a lower DOOR-RADAR score compared to a patient of the CG, whose anti-infective treatment is guided by standard of care microbiological analyses only.

#### Secondary outcomes

**Fig. 1:**
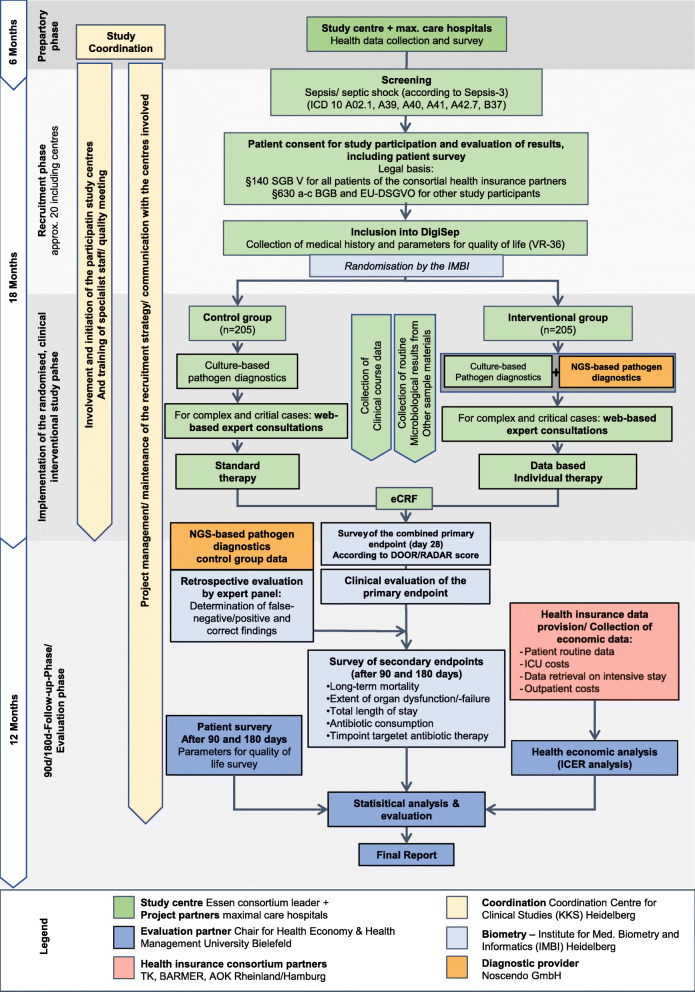
Detailed flow chart of specific procedures, assessments and visits of *DigiSep*-trial

**Fig. 2:**
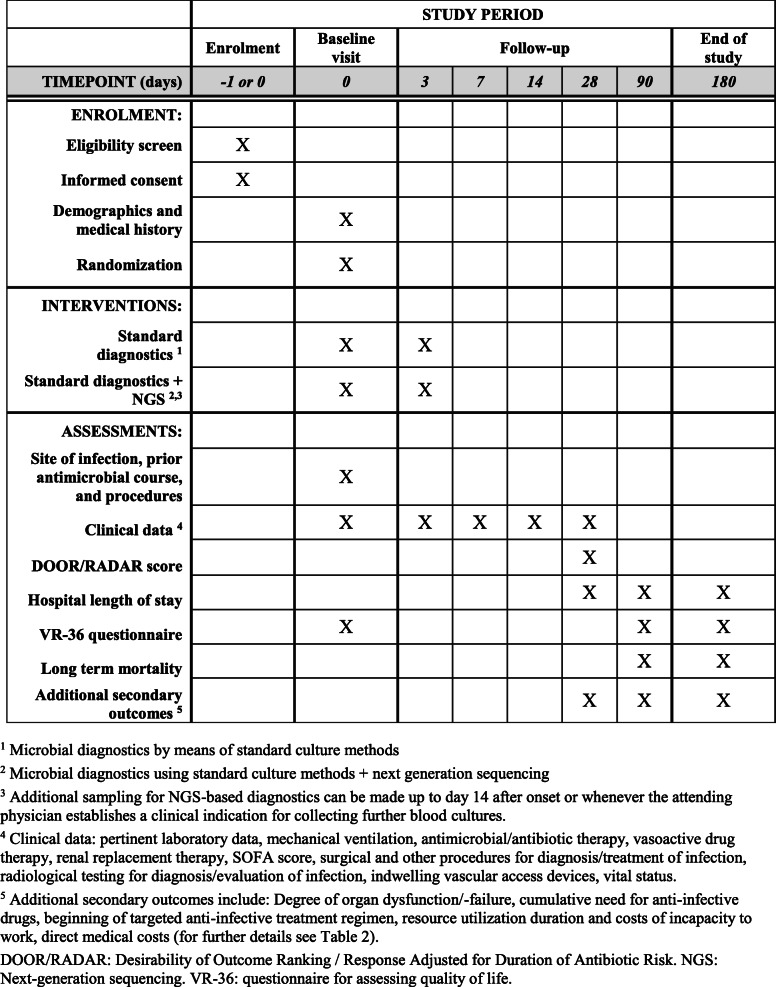
Schedule of enrolment, interventions, and assessments (SPIRIT figure)

A detailed description of all secondary outcomes is given in Table 2. As part of secondary outcomes, the cost-effectiveness of the new procedure will be evaluated. It is postulated that the length of hospital stay, long-term mortality, incidence of organ dysfunction/failure, the duration of anti-microbial therapy as well as the costs of complications and outpatient aftercare will be reduced. Moreover, a significant improvement in the QoL of affected patients is expected. A detailed flow chart of all trial specific procedures, assessments, and visits for participants is provided in Fig. 1. In Fig. 2, the timing of each visit as well as the procedures and assessments performed at each visit are displayed.

### Sample size calculation

For sample size calculation, a sophisticated secondary outcome analysis of the following four large sepsis studies of the SepNet Study Group (VISEP [15], MAXSEP [16], HYPRESS [17], and SISPCT [18]) was performed, in which the above described OCO-groups were represented as follows: OCO 1: 33%, OCO 2: 3%, OCO 3: 22%, OCO 4: 14%, and OCO 5: 28%. It is assumed that the anti-infective treatment period in OCO 1 and OCO 2 is 7 days in average, whereas it is prolonged up to 15 days (with a standard deviation of 10 days) in average in OCO 3 and OCO 4. Based on the assumption that the OCO group can be improved by at least 2 stages in 42% of IG patients in parallel to a shortening of the anti-infective treatment by 2 days, this would result in a power of 90% with 180 patients in each of the two study groups (CG, IG), provided that an IG patient has a lower DOOR/RADAR score as compared to a CG patient with a relative effect of *p* = 0.6. Including an additional drop-out rate of 12.5% (and considering these patients as OCO 5) would finally result in an overall sample size of 410 patients (CG: *n* = 205/IG: *n* = 205).

### Recruitment process

All adult patients (≥ 18 years) in the participating centers with suspected or proven sepsis/septic shock will be considered for inclusion in this study.

### Data collection methods

All data collected in this trial will be recorded on standardized electronic case report forms (eCRF: REDCap/URL: www.project-redcap.org). The investigators are responsible for full documentation of patient data required by the study protocol and for ensuring that all parts of the eCRFs are filled in correctly.

### Data management

All protocol-required information collected during the trial must be entered by the investigator, or designated representative, in the eCRF. The investigator, or designated representative, should complete the eCRF as soon as possible after information is available, preferably on the same day that a trial subject is seen for a trial procedure. Any outstanding entries must be completed as soon as possible. The completed eCRF must be approved by the investigator or by a designated sub-investigator. The approved eCRF is then sent to IMBI for data management. To ensure that the database reflects the eCRFs correctly, the IMBI accomplishes a double entry of data to the statistical program SAS. IMBI representatives will check completeness, validity, and plausibility of data using validating programs that generate queries when indicated. All validation rules will be predefined in a data validation plan. The investigator or the designated representatives are obliged to clarify or explain the queries. If no further corrections are to be made in the database, it will be closed and used for statistical analysis. The data will be managed and analyzed according to the appropriate standard operation procedure (SOPs) valid in the IMBI. For additional health economic analyses, IMBI and all participating health insurance companies will provide the Chair of Health Economics and Health Care Management, Bielefeld University, with pseudonymized eCRF and health insurance data via a special trust agency. Apart from health economic aspects, the Chair of Health Economics and Health Care Management, Bielefeld University, will assess the QoL of all participating patients using the VR-36 questionnaire [13]. According to §13 of the GCP Ordinance [19], all important trial documents (e.g., CRFs) are archived for at least 10 years after completion of the clinical trial.

### Statistical methods

The primary hypothesis will be analyzed using Mann-Whitney *U* test. Primary analysis will include all randomized patients (intention to treat (ITT)-population). Although DOOR/RADAR score values can be calculated reliably, any missing data will be replaced with multiple imputation methods [20]. The imputation model will be based on the variables age, SOFA score at sepsis onset, and survival and furthermore assumes that missing values of the primary endpoint are “missing at random”. Accordingly, missing values can be estimated by relevant co-variables in the imputation model. In order to review the assumptions of the imputation model, sensitivity analyses as well as “best case” and “worst case” scenarios will be performed. Moreover, these cases will be analyzed without the missing variables (complete case analysis). The primary endpoint will additionally be analyzed in a dichotomous way (good vs. bad outcome: e.g., OCO 1 versus OCO 2-5) using a logistic regression model, including group membership (CG, IG) and additional confounders (e.g., age, sex, SOFA score, comorbidities) as co-variates. The primary as well as all secondary endpoints will be presented using descriptive statistical methods. Moreover, 95% confidence intervals as well as descriptive *p* values for the effect estimates will be reported. In addition, mortality at 90 and 180 days will be evaluated using methods for survival-time analyses (Kaplan-Meier, Cox-proportional-Hazards-Regression). Wherever possible, graphs are used for visualization. All statistical tests will be performed using SAS (SAS Institute, Cary, NC). A *p* value of < 0.05 will be considered statistically significant. Detailed information of all statistical methods will be summarized and refined in a detailed statistical analytic plan (SAP).

### Data monitoring

Monitoring will be done at least twice in each study center (shortly after the inclusion of the first patient and at the end of the study) by personal visits from a clinical monitor of the Coordination Centre for Clinical Trials (KKS), Ruprecht-Karls-University, Heidelberg. The monitor will check patients´ informed consent and review the entries in the eCRFs on the basis of source documents. The investigator must allow the monitor to verify all essential documents and must provide support at all times. By frequent communication (letters, telephone, or email), the site monitor will ensure that the trial is conducted according to the protocol and regulatory requirements.

### Harms

Adverse events (AE) are restricted to complications of study-related blood draws (local lesions at puncture site or volume of blood draws). Such minor AEs are recorded in the eCRF. Serious AEs (SAE) resulting in death, a life-threatening state, a prolongation of existing hospitalization, a persistent or significant disability or incapacity due to study participation are not expected. This is due to the fact that standard-of-care diagnostics and treatment are guaranteed in both study groups, with the only difference that additional information resulting from NGS-based digital precision diagnostics are made available in IG patients.

### Ethics

Described procedures are meant to ensure that all parties involved abide by the principles of Good Clinical Practice (GCP) [19, 21] and those stipulated in the Declaration of Helsinki [22]. The study will be conducted in accordance with local statutory and implementing provisions.

### Research ethics approval

Prior to the beginning of the clinical trial, the study protocol, the patient information and informed consent, and all other required documents will be submitted to the competent ethical review committees of all participating centers. A first positive ethical vote has been given by the Ethics Committee of the Medical Faculty of the University Duisburg-Essen (Trial Code No. 20-9352-BO).

### Consent or assent

The members of the study group must inform eligible patients, both orally and in writing in an intelligible form about nature, significance, and implications of the study. Before participants can be enrolled in *DigiSep*, they must consent to participation in writing. For potential trial participants who are incapable, the following procedure applies: If a legal guardian exists, they are duly informed in accordance with the regulations and subsequently consent to participation in writing. If no legal guardian exists, participants are enrolled in the clinical trial after a near family member has been informed about nature, significance, and implications of the trial and has agreed to participation in the study mindful of the interest of the patient concerned (also by telephone). In summary proceedings, the designation of a legal guardian is begun at the district court. If no near family member is available, participants are enrolled after a guardianship judge or an independent medical consultant has been informed about nature, significance, and implications and has agreed to participation in the study mindful of the interest of the patient concerned (also by telephone). A near family member is appointed legal guardian earliest possible; they are duly informed in accordance with the regulations and subsequently consent to participation in writing (delayed consent). In any case, informed consent of study participants is sought retrospectively once they are capable of giving consent again.

### Justification for enrolment of participants not capable of giving consent

Bloodstream infections remain one of the major challenges in intensive care medicine, leading to sepsis or even septic shock in many cases. Due to the lack of timely diagnostic approaches with satisfying performance characteristics, mortality rates of sepsis are still unacceptably high. However, a prompt diagnosis of the causative microorganism is critical to improve outcome of bloodstream infections. Although various targeted molecular tests for blood samples are available, time-consuming blood culture-based approaches represent the international gold standard for identifying the underlying pathogen. With regard to these alarming figures, the presented clinical trial (*DigiSep*) is designed to investigate a comprehensive diagnostic approach, including NGS-based digital precision diagnostics, standard-of-care microbiological analyses, and facultative expert discussions. The majority of affected patients are sedated and given artificial ventilation. Even before being sedated, affected patients may be incapable of giving consent due to the underlying severe infection, inflammatory response, and severe pain. Therefore, in these cases, informed consent to participate in *DigiSep* needs to be given by a legal guardian until the affected patient is capable of consent. Nevertheless, especially these critically ill patients need to be enrolled in *DigiSep*, in order to assess the diagnostic value of the above described holistic and comprehensive approach for early detection of the causative microorganism in sepsis. This might help to improve outcome of patients suffering from sepsis due to an early optimization of the anti-infective treatment regime. This might especially be true for patients where classic microbiological or molecular diagnostic approaches fail.

### Confidentiality

Data collected are handled in accordance with the provisions of the Federal Data Protection Act (BDSG) [23]. During the clinical trial, participants are solely identified by a distinct reference number. For storage on a computer, the provisions of the BDSG [23] are abided by. Data are handled with strict confidentiality. For protection of these data, organizational measures are taken to prevent disclosure to unauthorized third parties. The relevant rules of the country-specific data legislation are complied with.

### Protocol amendments

Changes to the protocol are made in writing and require the approval of all signatories of the protocol. Subsequent amendments also require a positive assessment from the competent ethics committee.

### Dissemination

The *DigiSep* trial is planned to be reported in scientific peer-reviewed journals. The results will also be presented at relevant scientific conferences and symposiums. All contributors to the study will be offered authorship if they fulfill the International Committee of Medical Journal Editors (ICMJE) recommendations for authorship. No professional writers will be used.

## Discussion

Despite an underlying infection, positive blood cultures revealing the causative pathogen can only be obtained in a minority of septic patients [16, 24, 25]. This is at least partially attributable to technical shortfalls of blood culture processing. Moreover, the presence of strictly localized foci, fastidious organisms or very low rates of viable microorganisms in blood stream increase the risk of false-negative blood culture results [26]. Due to this diagnostic dilemma, culture-independent molecular diagnostic procedures, such as polymerase chain reaction (PCR)-based techniques, have been developed for identifying the causative pathogen in infected patients [27–31]. However, due to technical limitations of these PCR-based approaches (e.g., inability to quantify patients´ bacterial load in blood stream, restricted detection of antibiotic resistance markers, restricted pathogen detection depending on the included primers (= targeted approach), increased occurrence of contradictory or ambiguous results, low positive predictive value), this technique also appears to be far from perfect and has therefore not been widely applied in daily clinical routine [7]. A new molecular diagnostic concept of unbiased sequence analyses of circulating cell-free deoxyribonucleic acid (cfDNA) in human plasma samples by NGS revealed promising results and might therefore be a suitable diagnostic tool for critically ill patients suffering from bloodstream infections [9, 11]. As compared to PCR-based assays, NGS allows for a data-driven diagnosis without the need for specific primers (= open approach) and has the potential to detect bacterial, fungal and viral pathogens as well as parasites in one single assay. Moreover, by establishing a proprietary statistical framework, this new NGS-based approach enables a reliable differentiation between the disease-causing pathogen and potential microbial contaminants (e.g., coagulase-negative staphylococci). This goes far beyond state-of-the-art molecular approaches for the diagnosis of infecting organisms in septic specimens and might be especially useful for the diagnosis of cases where classic microbiological or molecular diagnostic approaches fail.

A potential limitation of the present study may be the design as an open-label trial without blinding. Blinding of the treating physicians, which in part belong to the data collectors, is not possible because they may have to interact with the anti-infective expert group whenever necessary. Moreover, the statisticians will not be blinded because they analyze data regarding group outcome. With respect to their group allocation patients may not be informed initially, but systematic blinding is not part of the protocol.

## Trial status

Protocol version: Version 2 (July 14, 2020)

Patient and data recruitment will be started as soon as the positive votes of all involved local ethic committees have been received. Recruitment is expected to be completed by June 2023.

## Supplementary Information

**Additional file 1: Supplemental File 1**: The SPIRIT 2013 Checklist.

**Additional file 2: Supplemental File 2**: Immunosuppressive host factors.

**Additional file 3: Supplemental File 3**: The SOFA score.

## Data Availability

The datasets generated and/or analyzed during the current study are available from the corresponding author on reasonable request.

## Abbreviations

AE: Adverse events
AKI: Acute kidney injury
BDSG: Federal data protection act
BGB: German civil code
BSI: Blood stream infection
cfDNA: Cell-free deoxyribonucleic acid
CG: Control group
eCRF: Electronic case report forms
DNA: Deoxyribonucleic acid
DOOR: Desirability of outcome ranking
DRKS: German clinical trials register
GCP: Good clinical practice
ICD: International classification of diseases
ICU: Intensive care unit
IG: Intervention group
IMBI: Institute for medical biometry and informatics
IMC: Intermediate care unit
ITT: Intention to treat
KKS: Coordination centre for clinical trials
LOS: Length of stay
MAP: Mean arterial pressure
NGS: Next-generation sequencing
OCO: Overall clinical outcome
PCR: Polymerase chain reaction
PCT: Procalcitonin
QoL: Quality of life
RADAR: Response adjusted for duration of antibiotic risk
RRT: Renal replacement therapy
SAE: Serious adverse event
SAP: Statistical analytic plan
SGB: Social security statute book
SOFA: Sequential organ failure assessment
SOP: Standard operation procedure
TIFOnet: Translational intensive care research network on organ dysfunction
