# Supplementary material for: Optimization of sepsis therapy based on patient-specific digital precision diagnostics using next generation sequencing (DigiSep-Trial)—study protocol for a randomized, controlled, interventional, open-label, multicenter trial
Source: Trials. 2021 Oct 18;22:714. doi: 10.1186/s13063-021-05667-x (PMC8522064; doi:10.1186/s13063-021-05667-x)
Supplement: Supplementary file 2 — Additional file 2: Supplemental File 2: Immunosuppressive host factors. [file 13063_2021_5667_MOESM2_ESM.docx]

**Supplemental File 2.** Immunosuppressive host factors ^12^.

| **Immunosuppressive host factors** |
| --- |
| Recent history of neutropenia (<0.5 × 10^9^ neutrophils/L [<500 neutrophils/mm^3^] for >10 days) temporally related to the onset of the disease |
| Receipt of an allogeneic stem cell transplant |
| Prolonged use of corticosteroids at a mean minimum dose of 0.3 mg/kg/day of prednisone equivalent for >3 weeks |
| Treatment with other recognized T cell immunosuppressants, such as cyclosporine, TNF-*α* blockers, specific monoclonal antibodies (such as alemtuzumab), or nucleoside analogues during the past 90 days |
| Inherited severe immunodeficiency (such as chronic granulomatous disease or severe combined immunodeficiency) |
| Abbreviations: TNF, tumor necrosis factor |
