# Supplementary material for: Optimization of sepsis therapy based on patient-specific digital precision diagnostics using next generation sequencing (DigiSep-Trial)—study protocol for a randomized, controlled, interventional, open-label, multicenter trial
Source: Trials. 2021 Oct 18;22:714. doi: 10.1186/s13063-021-05667-x (PMC8522064; doi:10.1186/s13063-021-05667-x)
Supplement: Supplementary file 3 — Additional file 3: Supplemental File 3: The SOFA score. [file 13063_2021_5667_MOESM3_ESM.docx]

**Supplemental File 3.** The SOFA-score ^2^.

| **SOFA-Score** | | | | | |
| --- | --- | --- | --- | --- | --- |
| **Parameters** | **Points** | | | | |
|  | **0** | **1** | **2** | **3** | **4** |
| Horowitz-Ratio [mmHg] | >400 | ≤400 | ≤300 | ≤200 | ≤100 |
| Thrombocyte count [10^3^/µl] | >150 | ≤150 | ≤100 | ≤50 | ≤20 |
| Bilirubin [mg/dl] | <1,2 | 1,2-1,9 | 2,0-5,9 | 6,0-11,9 | >12,0 |
| Arterial Hypotension | MAP >70mmHg | MAP <70mmHg | Dopamine ≤5µg/kg/min  o. dobutamine (dose-independ.) | Dopamine 5,1-15 µg/kg/min  o. epinephrine ≤0,1 µg/kg/min  o. norepinephrine ≤0,1 µg/kg/min | Dopamine >15 µg/kg/min  or epinephrine >0,1 µg/kg/min  or norepinephrine >0,1 µg/kg/min |
| GCS [points] | 15 | 13-14 | 10-12 | 6-9 | <6 |
| Creatinine [mg/dl] | <1,2 | 1,2-1,9 | 2,0-3,4 | 3,5-4,9 | >5 |
| Abbreviations: SOFA, Sequential Organ Failure Assessment Score; GCS, Glasgow Coma Scale; MAP, mean arterial pressure | | | | | |
